# Zika virus infection induces endoplasmic reticulum stress and apoptosis in placental trophoblasts

**DOI:** 10.1038/s41420-020-00379-8

**Published:** 2021-01-26

**Authors:** Philma Glora Muthuraj, Prakash K. Sahoo, Madison Kraus, Taylor Bruett, Arun S. Annamalai, Aryamav Pattnaik, Asit K. Pattnaik, Siddappa N. Byrareddy, Sathish Kumar Natarajan

**Affiliations:** 1 Department of Nutrition and Health Sciences, University of Nebraska-Lincoln, Lincoln, 68583-0806 NE USA; 2 Nebraska Center for Virology, University of Nebraska-Lincoln, Lincoln, NE USA; 3 School of Veterinary Medicine and Biomedical Sciences, University of Nebraska-Lincoln, Omaha, NE USA; 4 Department of Pharmacology and Experimental Therapeutics, University of Nebraska Medical Center, Omaha, NE USA; 5 Department of Genetics, Cell Biology and Anatomy, University of Nebraska Medical Center, Omaha, NE USA; 6 Department of Biochemistry and Molecular Biology, University of Nebraska Medical Center, Omaha, NE USA; 7 Child Health Research Institute, University of Nebraska Medical Center, Omaha, NE USA

**Keywords:** Viral infection, Apoptosis

## Abstract

Zika virus (ZIKV) infection to a pregnant woman can be vertically transmitted to the fetus via the placenta leading to Congenital Zika syndrome. This is characterized by microcephaly, retinal defects, and intrauterine growth retardation. ZIKV induces placental trophoblast apoptosis leading to severe abnormalities in the growth and development of the fetus. However, the molecular mechanism behind ZIKV-induced apoptosis in placental trophoblasts remains unclear. We hypothesize that ZIKV infection induces endoplasmic reticulum (ER) stress in the trophoblasts, and sustained ER stress results in apoptosis. HTR-8 (HTR-8/SVneo), a human normal immortalized trophoblast cell and human choriocarcinoma-derived cell lines (JEG-3 and JAR) were infected with ZIKV. Biochemical and structural markers of apoptosis like caspase 3/7 activity and percent apoptotic nuclear morphological changes, respectively were assessed. ZIKV infection in placental trophoblasts showed an increase in the levels of CHOP mRNA and protein expression, which is an inducer of apoptosis. Next, we also observed increased levels of ER stress markers such as phosphorylated forms of inositol-requiring transmembrane kinase/endoribonuclease 1α (P-IRE1α), and its downstream target, the spliced form of *XBP1* mRNA, phosphorylated eukaryotic initiation factor 2α (P-eIF2α), and activation of cJun N-terminal Kinase (JNK) and p38 mitogen activated protein kinase (MAPK) after 16–24 h of ZIKV infection in trophoblasts. Inhibition of JNK or pan-caspases using small molecule inhibitors significantly prevented ZIKV-induced apoptosis in trophoblasts. Further, JNK inhibition also reduced *XBP1* mRNA splicing and viral E protein staining in ZIKV infected cells. In conclusion, the mechanism of ZIKV-induced placental trophoblast apoptosis involves the activation of ER stress and JNK activation, and the inhibition of JNK dramatically prevents ZIKV-induced trophoblast apoptosis.

## Introduction

Zika virus (ZIKV) infection reports were sporadic in the African continent, but later gained significance due to 2015 endemic outbreaks. ZIKV was first isolated from a febrile macaque in the Ugandan Zika forest, and only few cases of human infections were reported in early 1940s. ZIKV infection during pregnancy is associated with congenital Zika virus syndrome (CZS)^1^. Infected infants manifest severe birth defects such as microcephaly, retinal defects, and intrauterine growth retardation (IUGR) leading to still births as well as increased infant mortality after 2 to 3 days postpartum^2^.

ZIKV is a single stranded positive sense RNA virus and its 10.8 kb genome can be directly translated into viral proteins. There are three structural proteins needed for assembly of viral particles, such as envelope, precursor membrane protein and capsid protein, and seven non-structural proteins (NS1, NS2A, NS2B, NS3, NS4A, NS4B, and NS5), which aid in replication of the virus and are involved in attenuating host innate immune response^3–6^. Virus size is roughly 50 nm with 180 copies of envelope and membrane proteins encompassing the outer structure. Envelope, the main structural protein covering the virus forms raft configuration with three protein dimers arranged in parallel manner^7^.

ZIKV infection is known to cause cell cycle arrest and apoptosis in progenitor neuronal cells, placental trophoblast, and Hofbauer cells^8–11^. Placental route plays a major role in disease transmission from the ZIKV infected mother to the fetus. The normal physiological role of the placenta, as a transient organ connecting the dam and the fetus is crucial for the fetal growth and survival through several functions like nutrient transport, respiratory gas exchange, and metabolism of waste products^12^. Stromal cores in the chorionic villi includes the fetal blood vessels and Hofbauer cells (placental resident macrophages), which regulate the branching of the villous^13^. Recent studies suggest that placental epithelial cells and the other placental cells, such as endothelial cells and Hofbauer cells can also be infected and results in dissemination of ZIKV from mother to fetus^14–16^. ZIKV was found to actively replicate in placental fibroblasts and Hofbauer cells resulting in spread of the infection to the neuronal progenitor cells of the fetal brain^17^. Further, T cell immunoglobulin and mucin domain 1 (TIM1)^18^, which is considered as important cofactor for the entry of ZIKV into cells is highly expressed in cytotrophoblasts, invasive cytotrophoblasts, placental fibroblasts, umbilical vein endothelial cells, hofbauer cells, and amniochorionic membranes^16,19^. Other cofactors such as Tyro3 and Axl^18,20^, implicated in ZIKV entry are also variably expressed in the placental cells^16^. In the present study, we show evidence for ZIKV infection to placental trophoblast results in ER stress and apoptosis via MAPK activation.

## Results

### Zika virus infection induces placental trophoblast apoptosis

**Fig. 1:**
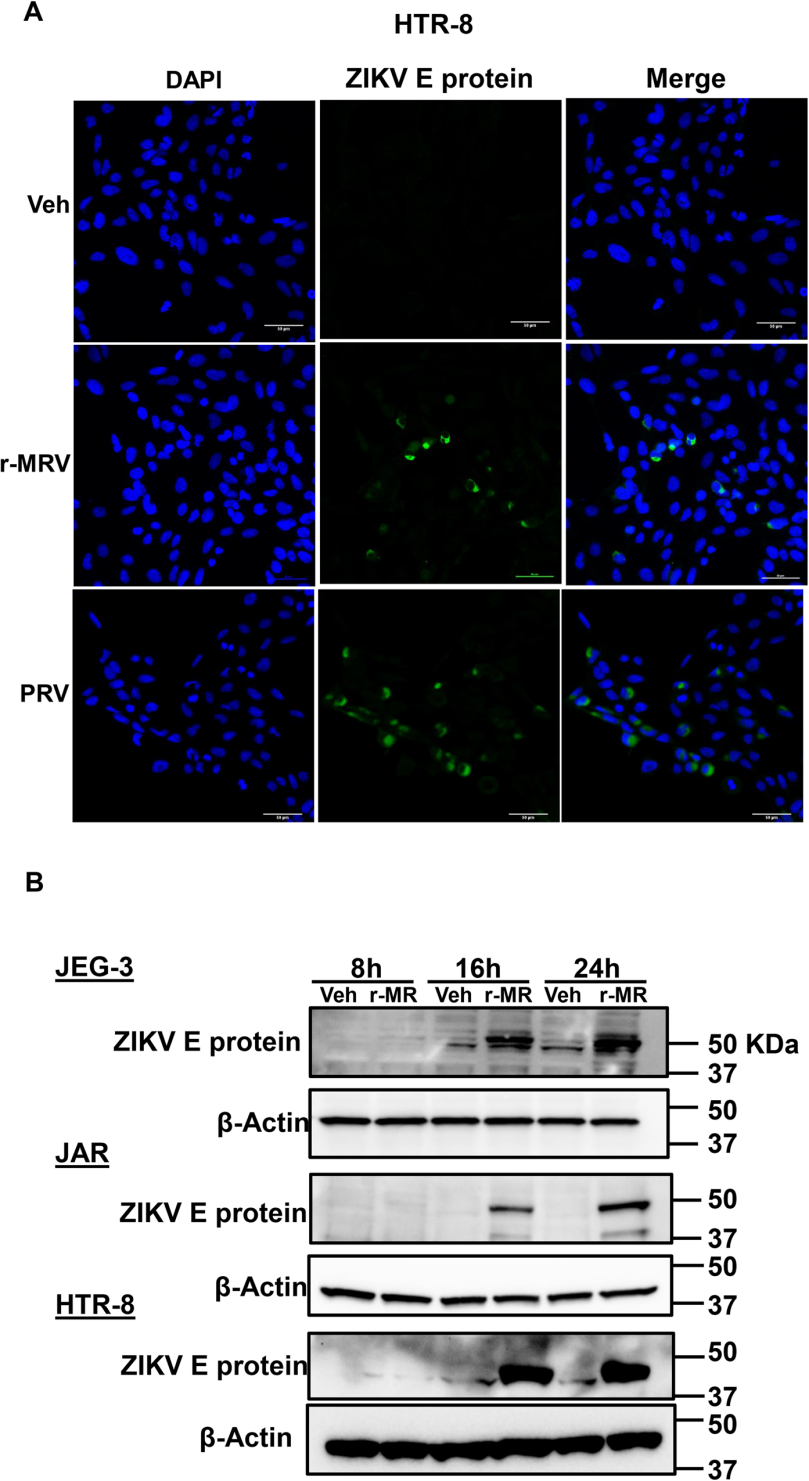
ZIKV infects placental trophoblasts. **a** Immunofluorescence staining of Viral E protein in HTR-8 (HTR-8/SVneo) cells 72 h postinfection with 0.1 multiplicity of infection (MOI) recombinant MR strain (r-MRV) or PRVABC-59 (PRV) or uninfected vehicle cells. Nuclei were stained with DAPI and ZIKV E protein stained with rabbit polyclonal primary antibody and Alexaflour 488-conjugated ant-rabbit secondary antibody. **b** Western blot analysis showing increased expression of viral E protein, 16–24 h postinfection when compared to uninfected vehicle cells in JEG-3, JAR, and HTR-8. β-Actin was used as a loading control.

**Fig. 2:**
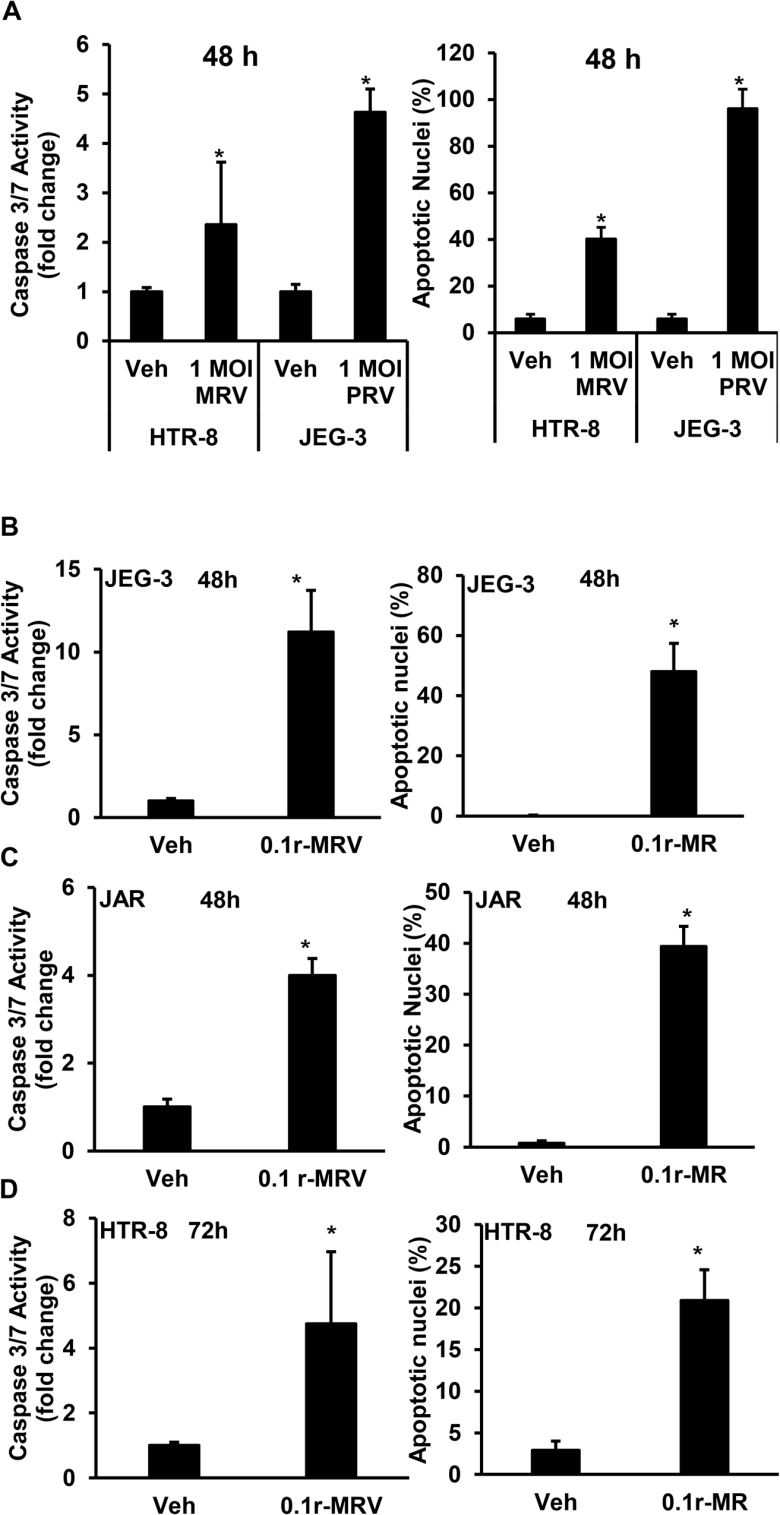
ZIKV infection induces placental trophoblast apoptosis. **a** JEG-3 and HTR-8 infected with 1.0 MOI MRV or PRV showing significant increase in caspase 3/7 activity compared to uninfected vehicle cells (left panel). Similarly, ZIKV infection with 1.0 MOI MRV or PRV also showed increased percentage apoptotic nuclei compared to uninfected vehicle cells (right). Similarly, ZIKV infection with 0.1 MOI r-MRV to placental trophoblast also induces apoptosis as evidenced by an increase in caspase 3/7 activity and percent apoptotic nuclei and in JEG-3 (**b**), JAR (**c**), and HTR-8 (**d**) cells compared to uninfected vehicle cells. Each value presents mean ± SEM of biological replicates (*n* = 4) for caspase 3/7 activity and (*n* = 3) for percentage apoptotic nuclear morphology, *P < 0.05 compared to uninfected vehicle cells; statistical comparison by Student’s *t*-test.

We performed immunofluorescence analysis in placental trophoblasts (HTR-8 cells) infected with 0.1 MOI of r-MRV or PRV strain of ZIKV. We observed viral E protein staining after 72 h in ZIKV infected trophoblasts, while there was no staining observed in vehicle or mock infected trophoblast cells (Fig. 1a). We also analyzed the expression of viral E protein after 8–24 h of infection in JEG-3, JAR, and HTR-8 cells. Viral E protein levels were dramatically increased at 16 or 24 h postinfection of ZIKV in JEG-3, JAR, and HTR-8 cells compared to mock infected cells. The levels of beta-actin were used as control and remains unchanged at different timepoints of infected cells and mock infected trophoblasts (Fig. 1b). Next, we assessed biochemical characteristics of apoptosis such as caspase 3 and 7 activation and nuclear morphological changes with ZIKV infection in placental trophoblast cells. Both first trimester-derived trophoblast (HTR-8) and term-derived trophoblast cells (JEG-3) cells infected with 1.0 MOI of MRV (MR766) and PRV strains of ZIKV, 48 h postinfection showed a significant increase in caspase 3 and 7 activity and percent apoptotic nuclei indicating apoptosis (Fig. 2a). We also assessed trophoblast apoptosis with 0.1 MOI of r-MRV strain after 48–72 h of infection and, we observed a significant increase in caspase 3/7 activity and percent apoptotic nuclei in placental trophoblasts (JEG-3, JAR, and HTR-8) (Fig. 2b–d). We also observed a trend in downregulation of anti-apoptotic Bcl_2_ protein with 0.1 MOI of r-MRV strain of ZIKV 16–24 h postinfection in JEG-3 and HTR-8 cells (Fig. S1). These data suggest that ZIKV infection of placental trophoblast cells induces apoptosis.

### ZIKV infection in trophoblasts induces CHOP nuclear translocation, a transcription factor that can activate apoptosis

**Fig. 3:**
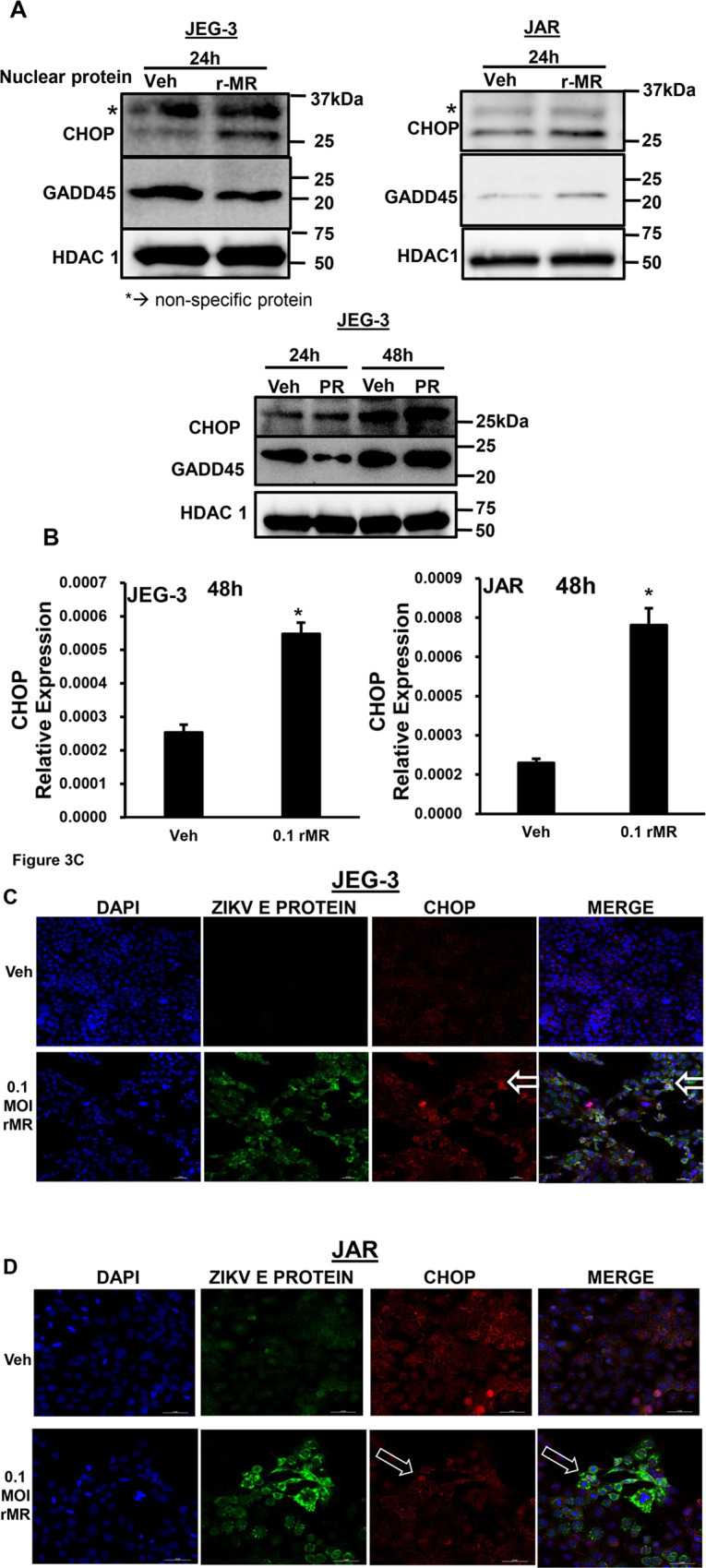
ZIKV infection induces the expression CHOP, a transcription factor that activate apoptosis. **a** Immunoblot analysis showed an increase in the expression of CHOP in nuclear extracts of ZIKV infected cells (0.1 MOI r-MRV) compared to vehicle uninfected cells in JEG-3 (left) and JAR cells (right), increased nuclear levels of CHOP can act as an initiator for apoptosis. JEG-3 (left) and JAR cells (right). We also observed increased GADD45 levels in nuclear protein, which is produced in response to DNA damage, after 24 h of infection in JAR cells (right) with 0.1 MOI of r-MRV compared to uninfected vehicle cells and this increase of GADD45 in the infected cells was absent in JEG-3 (left). HDAC1 was used as a loading control. We also observed increase in expression of CHOP nuclear protein both 24 and 48 h postinfection and GADD45 nuclear protein around 48 h postinfection in JEG-3 cells with 0.1 MOI PRV (lower middle panel). **b** JEG-3 (left) and JAR (right) cells after 48 h of 0.1 MOI of r-MRV showed significant increase in CHOP mRNA levels compared to uninfected vehicle cells. CHOP mRNA expression were reported relative to 18 S rRNA. Each value presents mean ± SEM of biological replicates (*n* = 3), *P < 0.05 compared to uninfected vehicle cells; statistical comparison by Student’s *t*-test. **c** Immunofluorescence analysis was also performed to test CHOP nuclear translocation by using Viral E protein staining indicated as green signal and CHOP as red signal. JEG-3 cells: uninfected vehicle cells showed no viral E protein staining but has endogenous CHOP expression. Cells infected with 0.1 MOI r-MRV show nuclear CHOP expression surrounded by viral E protein staining in the cytoplasm in the merged panel. **d** JAR cells: uninfected vehicle cells show no viral E protein staining but endogenous CHOP expression is seen. Cells infected with 0.1 MOI r-MRV showed increased nuclear CHOP expression surrounded by viral E protein staining in the cytoplasm showed in the merged panel indicated in white arrow. Scale bar represents 50 µM size.

**Fig. 4:**
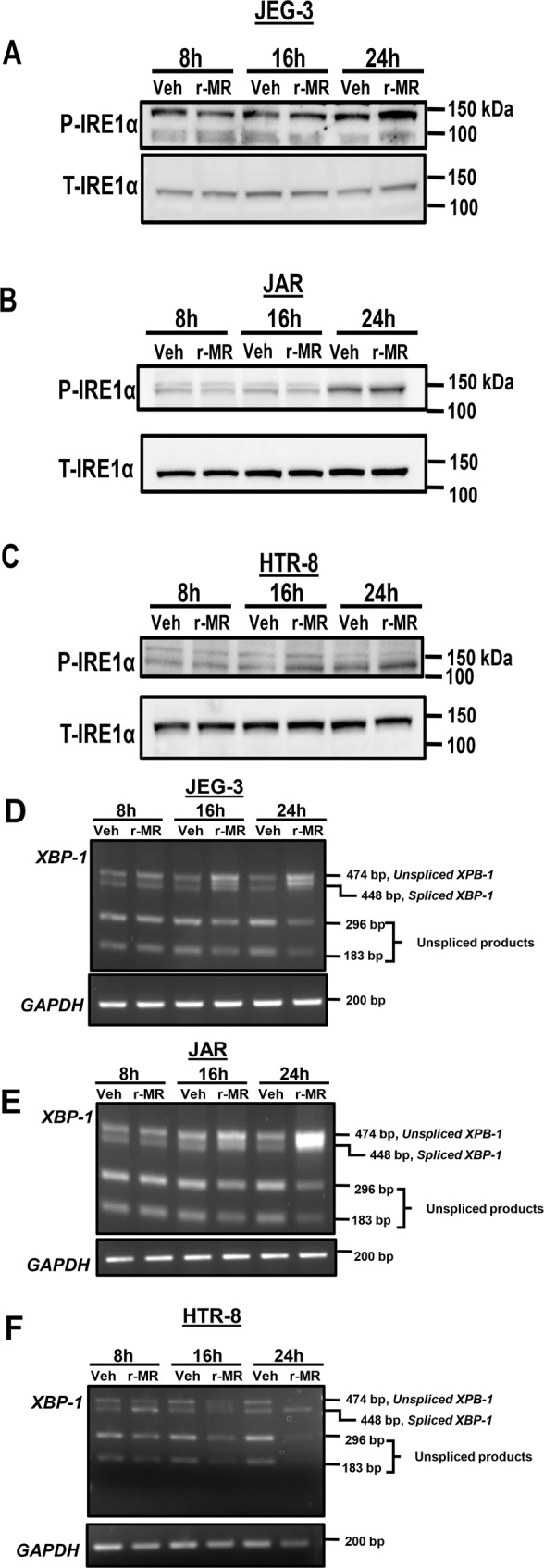
ZIKV infection activates IRE1α arm of ER stress in placental trophoblasts. **a** Western blot images shows increased levels of phosphorylated IRE1α after 24 h of infection with 0.1 MOI r-MRV in JEG-3 cells. **b** A slight increase in phospho-IRE1α was observed in JAR cells after 24 h of infection and HTR-8 cells also showed increased phospho- IRE1α after 16–24 h of ZIKV infection compared to uninfected vehicle cells. Trophoblasts were infected with 0.1 MOI r-MRV and XBP1 splicing was observed after 8, 16, and 24 h of infection. **c** JEG-3 cells showed an increased spliced form of *XBP1* mRNA at 24 h postinfection. **d** JAR cells infected with 0.1 MOI r-MRV strain showed increased levels of spliced XBP1 at 24 h of postinfection compared to mock-infected or uninfected vehicle cells. **e** HTR-8 cells infected with 0.1 MOI, r-MRV showed increased XBP1 spliced form starting from 8 h of postinfection and stayed elevated until 24 h of postinfection. The unspliced XBP1 cDNA is cleaved by PstI restriction enzyme and shows faster migration pattern than the spliced form. GAPDH was amplified as a loading control.

Sustained ER stress can progress the cells into apoptosis either via CHOP or JNK activation^25^. Active and spliced *XBP1* can trancriptionally increase expression of ER stress response proteins like CHOP^26^. We examined whether ZIKV infection can induce the expression of other downstream targets that activates apoptosis. We tested the nuclear translocation of CHOP with 0.1 MOI r-MRV ZIKV infection in trophoblasts and confirmed that there was increased nuclear translocation of CHOP protein after 24 h ZIKV infection in JEG and JAR cells (Fig. 4a). Similarly, there was a trend in increase in the levels of CHOP mRNA, 24 h postinfection in both JAR and JEG-3 cells where as it was 8 h postinfection in HTR-8 cells (Fig. S2). We also observed a significant increase in CHOP mRNA expression 48 h postinfection compared to uninfected vehicle cells in both JEG-3 and JAR cells (Fig. 4b). We further confirmed CHOP nuclear transclocation in JEG-3 (Fig. 4c) and JAR cells (Fig. 4d) using immunofluorescence analysis. We observed enhanced CHOP nuclear localization surrounded by viral E protein staining in the cytoplasm of r-MRV infected cells at 48 h postinfection. We also examined whether ZIKV infection induces Growth Arrest and DNA Damage-inducible 45 (GADD45) which can activate and promote apoptosis. Interestingly, we found that the nuclear levels of GADD45 were increased after 24 h of ZIKV infection in JAR cells (Fig. 3a). Further, JEG-3 cells infected with PRV 24–48 h postinfection showed increased nuclear translocation of CHOP (Fig. 3a, bottom panel). However nuclear levels of GADD45 were increased only after 48 h of infection with PRV, when compared to uninfected vehicle cells (Fig. 3a, bottom panel). These data suggests that both MRV and PRV similarly induced CHOP and GADD45 nuclear translocation.

### ZIKV infection induces ER stress in placental trophoblasts

**Fig. 5:**
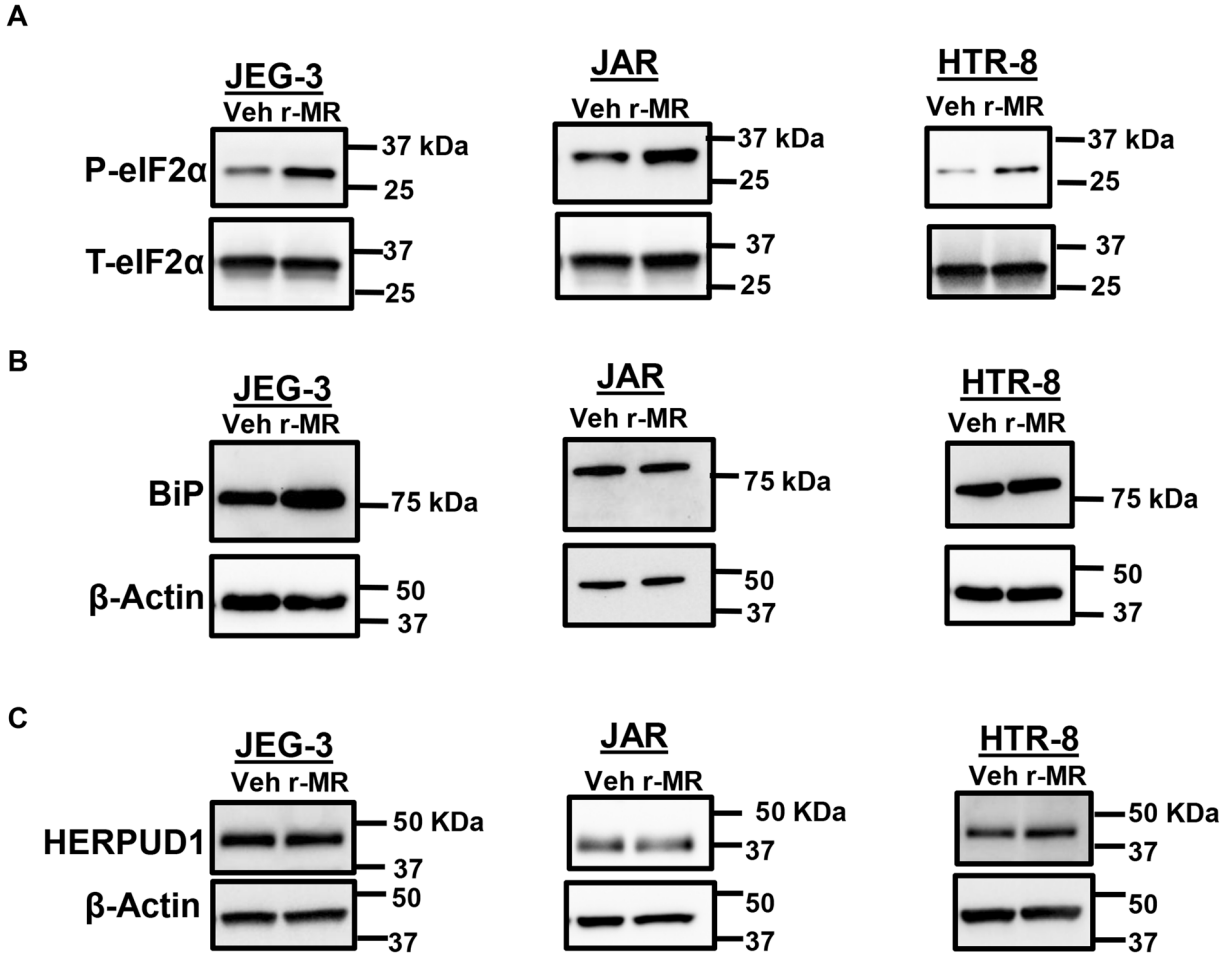
ZIKV infection and ER stress markers in trophoblasts. **a** Western blot analysis showing increase in the expression of phosphorylated eIF2α (P-eIF2α) after 24 h of infection with 0.1 MOI r-MRV in both JEG-3 and HTR-8 (HTR-8 SV/neo) cells and 16 h postinfection in JAR cells compared to uninfected vehicle cells. Total eIF2α levels remained constant. **b** ZIKV infected trophoblast also showed an increase in the levels of Bip/GRP78 in JEG-3 but not in JAR and HTR-8 cells compared to uninfected vehicle cells. **c** HERPUD1 levels were unchanged in uninfected and ZIKV infected JEG-3, JAR, and HTR-8 cells. Actin was used as a loading control.

We examined the activation of IRE1α arm of ER stress with ZIKV infection in placental trophoblasts. We observed an increase in the levels of phosphorylated IRE1α in placental trophoblasts after 24 h of ZIKV infection, suggesting that the ER stress sensor proteins are activated in JEG-3, JAR, and HTR-8 (Fig. 4a–c). However, the levels of total IRE1α protein were unchanged (Fig. 4a–c). Activated IRE1α, an endoribonuclease is known to enhance the splicing and processing of *XBP1* mRNA. We next examined the downstream target of activated IRE1α that is enhanced splicing of *XBP1* mRNA. We observed a dramatic increase in the levels of *XBP1 mRNA* splicing 8–24 h postinfection in JEG-3, JAR, and HTR-8 cells (Fig. 4d, e). JEG-3 and JAR cells showed a time dependent increase in the active spliced form of *XBP1* which was dramatically increased in 24 h. In HTR-8 cells, there was active form of spliced *XBP1* particularly evident at 8 h postinfection (Fig. 4f). GAPDH was used as loading control. ER stress has been shown to block global protein translation^27^. Host immune defense such as antiviral response by interferons are also known to activate eIF2α via phosphorylation^28^. We assessed the activation of eIF2α via phosphorylation. ZIKV infection of placental trophoblast showed increased levels of phosphorylated eIF2α (P-eIF2α) after 24 h of infection in JEG-3 and HTR-8 cells, whereas activation of eIF2α was observed after 16 h of infection in JAR cells and the levels of total eIF2α remain unaltered (Fig. 5a). Increased P-eIF2α levels can block global protein translation in the placental trophoblasts with ZIKV infection. We also found that Bip/GRP78, regulator of ER stress is slightly increased 24 h of postinfection in JEG cells, but this increase did not correspond to JAR and HTR-8 cells with ZIKV infection (Fig. 5b). Homocysteine Inducible ER Protein with ubiquitin like domain 1 (HERPUD1) expression also remained unchanged in both vehicle and r-MRV infected JEG-3, JAR, and HTR-8 cells (Fig. 5c).

### Activation of mitogen activated protein kinase (MAPK)

**Fig. 6:**
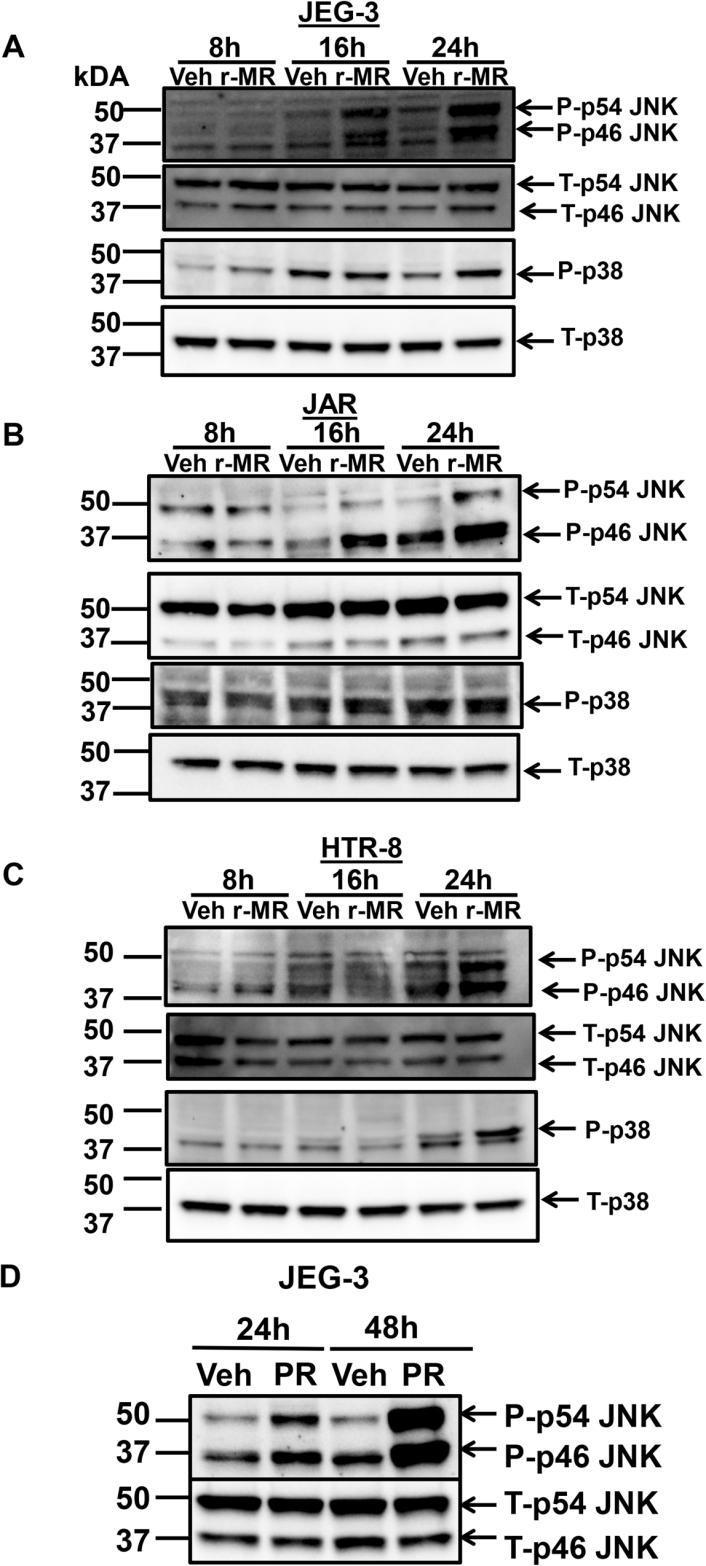
Activation of mitogen activated protein kinase (MAPK) in ZIKV infected trophoblasts. **a** Immunoblot analysis of JNK and p38 activation via phosphorylation were observed 8–24 h after infection with 0.1 MOI r-MRV in JEG-3 cells showed increase in phosphorylation of p54 and p46 components of JNK and phosphorylation of p38 MAPK as well compared to uninfected vehicle cells. Similarly, JNK and p38 activation via phosphorylation was also observed in JAR cells (**b**) and HTR-8 (**c**) 24 h of postinfection with 0.1 MOI r-MRV compared to uninfected vehicle cells. Total JNK and Total p38 levels were mostly unaltered between ZIKV infected and vehicle uninfected cells. **d** Activation of JNK was also seen with 0.1 MOI PRV 24–48 h of postinfection in JEG-3 cells compared to uninfected vehicle cells.

ER stress-induced activation of IRE1α has been shown to activate JNK^29,30^. To assess the mechanism of ZIKV-induced placental trophoblast apoptosis, we sought to test the activation of JNK, which can initiate apoptotic cell death during sustained ER stress. ZIKV infection in JEG-3 cells showed increased levels of JNK phosphorylation after 16–24 h of infection suggesting an activation of JNK (Fig. 6a). Similarly, we also observed an increase in phospho-JNK levels in JAR and HTR-8 cells, 24 h post infection in comparison to the total JNK levels (Fig. 6b, c). We also tested 0.1 MOI of PR strain infection in JEG-3 cells and observed increased phosphorylation of JNK, 24 and 48 h postinfection (Fig. 6d) when compared to uninfected vehicle cells. These data provide insights on the mechanism of ER stress and its contribution to the apoptotic signal. p38 MAPK is known have a regulatory control on CHOP^31^, also p38 activation in DENV is known to be associated with hepatic damage^32^. Here, we found that ZIKV infection also activates p38 MAPK via phosphorylation in JEG-3, HTR-8 cells after 24 h of infection (Fig. 6a, c) and in JAR cells it was more prominently after 16 h of infection (Fig. 6b). These data suggest that ZIKV infection induces p38 MAPK activation.

### Inhibition of JNK prevents ZIKV-induced trophoblast apoptosis

**Fig. 7:**
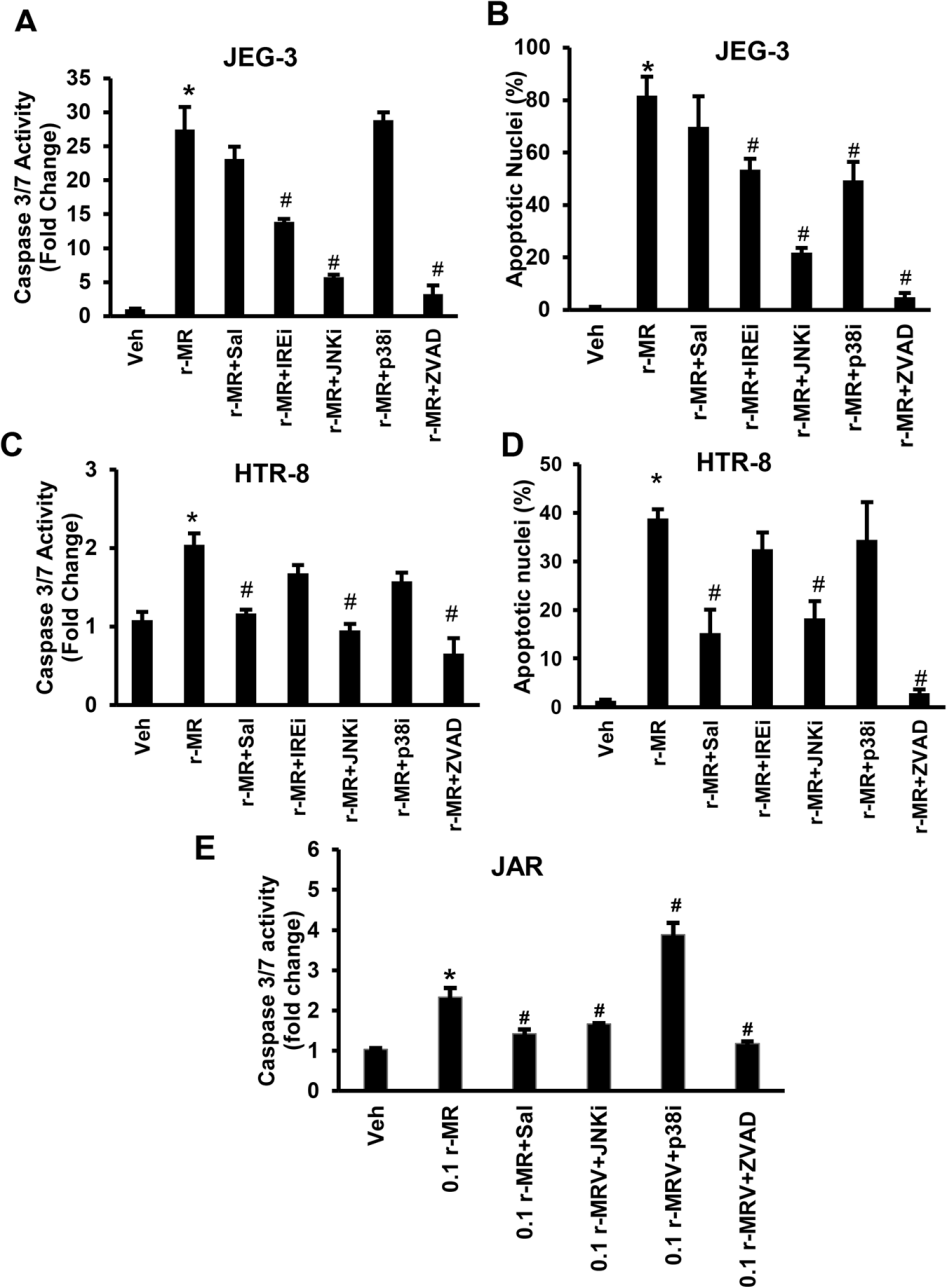
Inhibition of JNK prevents ZIKV-induced trophoblast apoptosis. **a**, **b** JEG-3 cells infected with 0.1 MOI of r-MRV for 48 h showed significant increase in apoptosis as evidenced by caspase 3/7 activity and percent apoptotic nuclear compared to uninfected vehicle treatment. ZIKV-induced placental trophoblast apoptosis were significantly prevented in JEG-3 cells infected with 0.1 MOI of r-MRV and IREi (STF-083010, IRE1α inhibitor), JNKi (SP600125, JNK inhibitor), or ZVAD (Z-VAD-fmk, pan caspase inhibitor) treatment, respectively. However, ZIKV infection and treatment of Sal (Salubrinal, eIF2α dephosphorylation inhibitor) did not prevent trophoblast apoptosis. **c**, **d** HTR-8 cells infected with 0.1 MOI of r-MRV for 72 h showed a dramatic increase in biochemical markers of apoptosis compared to uninfected vehicle cells. ZIKV-induced placental trophoblast apoptosis were significantly prevented in HTR-8 cells infected with 0.1 MOI of r-MRV and Sal (Salubrinal, eIF2α dephosphorylation inhibitor), JNKi (SP600125, JNK inhibitor), or ZVAD (Z-VAD-fmk, pan caspase inhibitor) treatment, respectively. However, ZIKV infection and IRE inhibition (STF-083010, IRE1α inhibitor) or p38 inhibition treatment does not prevent ZIKV-induced trophoblast apoptosis. **e** JAR cells infected with 0.1 MOI r-MRV 48 h postinfection showed activation of caspase 3/7 when compared to vehicle cells. Sal (Salubrinal, eIF2α dephosphorylation inhibitor), JNKi (SP600125, JNK inhibitor), or ZVAD (Z-VAD-fmk, pan caspase inhibitor) treatment in infected cells inhibited caspase 3/7 activation while p38 inhibition significantly increased caspase 3/7 activation. Each value presents mean ± SEM of (*n* = 4) for caspase 3/7 activity and *n* = 3 for percent apoptotic nuclei, *P < 0.05 compared to uninfected vehicle cells; ^#^*P* < 0.05 compared to ZIKV infected cells; statistical comparison by ANOVA with posthoc Bonferroni correction.

**Fig. 8:**
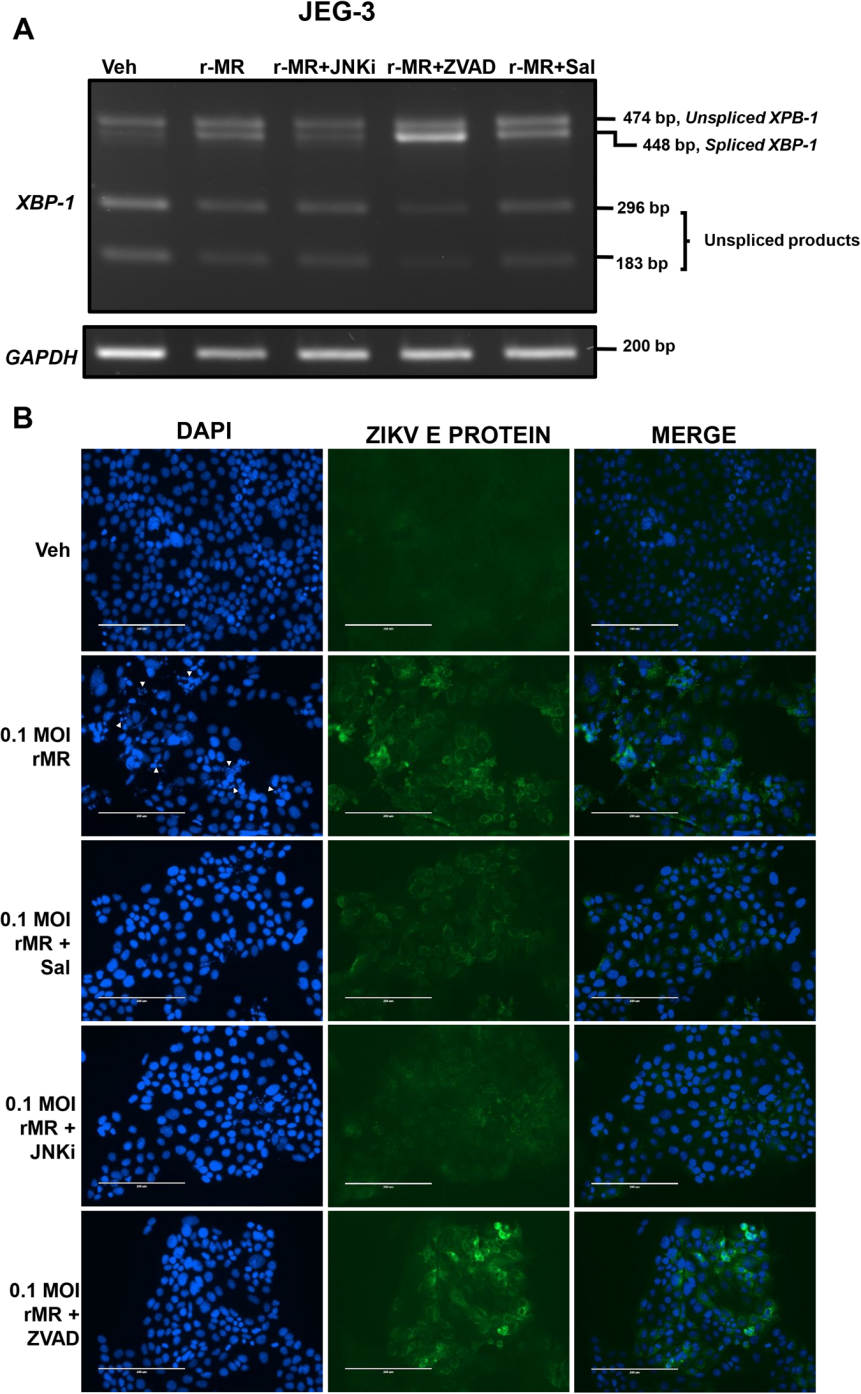
Inhibition of JNK reduces *XBP1* mRNA splicing and viral E protein. **a** JEG-3 cells showed an increase in the splicing of *XBP1* mRNA of the IRE1α arm of ER stress pathway with 0.1 MOI r-MRV, 48 h of postinfection compared to uninfected vehicle cells. *XBP1* mRNA splicing was reduced in the infected cells cotreated with JNKi (SP600125, JNK inhibitor). *XBP1* gene splicing were increased in infected cells cotreated with ZVAD (Z-VAD-fmk, pan caspase inhibitor), similarly a moderate increase in *XBP1* mRNA splicing were observed in Sal (Salubrinal, eIF2α dephosphorylation inhibitor) cotreatment to infected cells. **b** JEG-3 cells with 0.1 MOI r-MRV infection also showed viral E protein staining and fragmented nuclei in the DAPI staining compared to the uninfected vehicle cells. Treatment of JNKi (SP600125, JNK inhibitor) to r-MRV infected cells showed reduced E protein staining whereas the ZVAD (Z-VAD-fmk, pan caspase inhibitor) treated infected cells showed huge increase in viral E-protein staining with intact nucleus in the DAPI panel. The Sal (Salubrinal, eIF2α dephosphorylation inhibitor) treated infected cells also showed moderate viral E protein staining compared to r-MRV infected cells.

We used small molecule inhibitors of ER stress mediators like selective dephosphorylation inhibitor of eIF2α (Salubrinal, 20 µM), IRE1α inhibitor (STF-083010, 20 µM), JNK inhibitor (SP600125, 50 µM), p38MAPK inhibitor (SB203580, 50 µM), or pan caspase inhibitor (Z-VAD-FMK, 50 µM) to test the critical mediators of apoptosis. Similar to Fig. 1, ZIKV infection to trophoblast showed increased apoptosis as evidenced by dramatic increase in caspase 3/7 activity and percent apoptotic nuclei (Fig. 7). Treatment of ZIKV-infected trophoblast with SP600125 and Z-VAD-fmk showed a significant decrease in caspase 3/7 activation and percent apoptotic nuclei in trophoblasts infected with ZIKV. These results suggest that JNK and caspase activation are critical pathway that drive the ZIKV infected cells with persistent ER stress towards apoptosis (Fig. 7a–e). IRE1α inhibition using STF-083010 in JEG-3 cells showed partial protection against ZIKV-induced trophoblast apoptosis, however IRE1α inhibition does not significantly protect against ZIKV-induced apoptosis in HTR-8 cells (Fig. 7). In contrast, inhibition of ER stress using salubrinal significantly protected against ZIKV-induced apoptosis in HTR-8 cells. Treatment of salubrinal in JEG-3 cells showed a slight non-significant trend in protection against ZIKV-induced apoptosis (Fig. 7a). In JAR cells, caspase 3/7 activity was significantly inhibited when the infected cells were treated with salubrinal, or SP600125 or Z-VAD-fmk. In contrast, treatment of p38 MAPK inhibitor, SB203580 in ZIKV infected JAR cells showed significantly increased caspase 3/7 activation (Fig. 7e). These data suggest that activation of caspases and JNK are critical in ZIKV-induced trophoblast apoptosis. Similarly, *XBP1* mRNA splicing assay showed increased *XBP1 mRNA* splicing in JEG-3 with 0.1MOI r-MRV after 48 h of postinfection compared to uninfected vehicle cells (Fig. 8a). On the other hand, when the MRV infected cells treated with SP600125 (JNK inhibitor), we observed a dramatic reduction in the levels of XBP1 splicing. Interestingly, treatment of Z-VAD-fmk to the ZIKV infected cells showed dramatic increase in XBP1 splicing, and however treatment of salubrinal showed a moderate increase in XBP1 splicing compared to the 0.1rMRV infection alone (Fig. 8a). We then analyzed the expression of viral E protein using immunofluorescence in JEG-3 cells with ZIKV infection. There was a dramatic increase in viral E protein staining in 0.1r-MRV infected JEG-3 cells but was absent in uninfected vehicle cells. The DAPI panel shows fragmented nuclei in the 0.1 rMRV infected cells. ZIKV Infected cells treated with SP600125 (JNK inhibitor) showed dramatic reduction in the viral E protein staining. However, treatment of salubrinal showed moderate reduction in the viral E protein staining compared to the infected cells. Interestingly, treatment of Z-VAD-fmk in 01.rMRV infected cells showed enhanced viral E protein staining, and the nuclei in the DAPI panel are intact confirming the fact that Z-VAD-fmk has inhibited apoptosis suggesting that the viral particles are trapped inside the cells with the inhibition of caspase activity and are protected from viral release for further infection. These data suggests that ZIKV induces ER stress in a JNK-dependent manner and inhibition of JNK prevents ZIKV-induced ER stress and apoptosis.

## Discussion

The mechanism of ZIKV infection-induced trophoblast apoptosis is via activation of ER stress markers and JNK activation. The principal findings of this manuscript are: (1) ZIKV infection induces caspase-dependent apoptosis; (2) ZIKV infection induces three arms of ER stress; (3) Nuclear translocation of CHOP was observed in trophoblasts infected with ZIKV; and (4) Activation of JNK plays a critical role in ZIKV infection-induced trophoblast apoptosis. Even though initially it was thought that term placenta is resistant to ZIKV infection^33^ later it was found that ZIKV can cross the placental barrier by compromising the tight junctions^34^. Human placenta explant studies have also showed that there was an effective ZIKV replication in the placental tissues and trophoblast apoptosis^35^. There are also reports showing ZIKV replication in placental trophoblast cell lines^20,36,37^.ZIKV is known to activate innate immune pathways leading to inflammation in placental trophoblasts^20,37^. ZIKV infection can also cause placental trophoblasts to undergo apoptosis and release of new viral particles from the dead cells could facilitate ZIKV infection and spread to the growing fetus^38^. Interestingly, innate immune pathways can also stimulate apoptosis^39^. We also have observed caspase activation and characteristic apoptotic nuclear morphological changes in ZIKV infected trophoblasts, both first and third trimester-derived trophoblasts cells. ZIKV infected human fetal neural parenchymal tissue showed increased expression of Fas and Fas ligand suggesting that apoptosis contributes to fetal brain injury in ZIKV-induced microcephaly^40,41^. Our data suggest that there was slight decrease in anti-apoptotic proteins like Bcl2. Recently, ZIKV infection has been shown to delay apoptosis at early stages of infection to ensure the effective production of virions by regulating Bcl2^42^, this could be attributed to the reason that we could see only subtle changes in the Bcl2 levels after 8–24 h of infection, a time point where we observed changes in ER stress markers. These data suggest that ER stress precedes apoptotic events with ZIKV infection in trophoblasts. Further time course studies are required to measure the levels of pro-apoptotic and anti-apoptotic proteins with ZIKV infection-induced trophoblast apoptosis.

Flaviviruses are closely associated with ER stress as they replicate within the cellular membrane bound organelles, especially ER. Accumulation of structural and non-structural proteins in the ER results in the formation of convoluted spherules, which activates unfolded protein response (UPR). Apart from this, they also remodel ER in terms of its protein and lipid content^43,44^. Zika virus is a positive stranded RNA virus that has been shown to replicate in the ER membrane^44^. During viral infection, host cells activates UPR to combat the viral protein load in the ER membranes and induces ER stress and prolonged ER stress result in apoptosis^45,46^. Further, ZIKV infection to neural progenitor cells were also shown to upregulate ER stress and apoptosis and could results in the pathophysiology of ZIKV-associated microcephaly^47,48^. ER stress activates three different arms of the UPR, namely the PERK pathway, the IRE1α pathway, and the ATF6 pathway. There is always crosstalk among these pathways that orchestrates signals leading to survival or death of cells^49^.

Sustained ER stress and splicing of *XBP1* gene corresponds well to apoptosis^50,51^. IRE1α and XBP1 targets were also shown to be activated in ZIKV infected neuronal cells^52^. Although IRE1α and XBP1 activation is important for maintaining normal protein folding in the ER and biosynthesis of ER^53^. We observed increased apoptosis in placental trophoblasts with ZIKV infection along with increased *XBP1* splicing and IRE1α activation suggests that the mechanism of ZIKV-induced apoptosis could be due to the sustained ER stress^50,51^.

Sustained ER stress-induced IRE1 α activation has also been shown to activate MAP Kinases like JNK and p38 MAPK via phosphorylation. Sustained or prolonged ER stress is known to activate IRE1α which can result in the IRE1α dependent activation of JNK and p38 MAPK in association of apoptosis signal-regulating kinase 1 (ASK1) and TNF receptor associated factor 2 (TRAF2) can result in apoptosis^54,55^. Further, p38 MAPK is a critical regulator of pro-inflammatory cytokine and is found to be involved in ZIKV induced-inflammation of neuronal cells of the retina^56^. Further studies are underway to elucidate the critical role for p38 MAPK activation in inflammatory processes in placental trophoblasts with ZIKV infection.

Global protein translation shutdown was observed in ZIKV infected trophoblast as evidenced by enhanced phosphorylation of eIF2α suggesting PERK activation^57^. Double-stranded RNA-dependent protein kinase (PKR) also phosphorylates eIF2α, which is associated with antiviral interferon signaling pathway to keep viral replication under control^58^. Despite the potential to encounter a strong antiviral interferon response, ZIKV evades the innate immune system by modulating RIG-I (retinoic acid-inducible gene I) like receptor (RLR) signaling, thereby diminishing the effect of antiviral interferon response to the replicating ZIKV^59^. Increased levels of phospho-eIF2α is known to increase the formation of stress granules which composes preinitiation complexes and mRNA for its antiviral activity. However, ZIKV effectively evades this mechanism and uses the stress granule components to their advantage to aid their own replication^60,61^. Bip or GRP78 is a chaperone that binds to the ER membrane proteins IRE1α, PERK, and ATF6 in normal resting state, under ER stress it gets dissociated allowing the membrane proteins to be activated^62^. PERK phosphorylation leads to ATF4 transcription which in turn results in CHOP nuclear activation^63,64^. CHOP primes the cell for apoptosis by decreasing the expression of anti-apoptotic Bcl_2_ proteins and increasing expression of GADD45^65,66^. Our data suggests that ZIKV infection induces both CHOP and GADD45 levels in the nucleus. Further, GADD45 is known to be associated with regulation of apoptosis under stress or DNA damage^67^. Bip, a master regulator of ER stress is slightly increased 24 h of postinfection in JEG cells but this increase did not occur in JAR and HTR-8 cells with ZIKV infection. In a study involving Borna disease virus, purkinje cells showed CHOP induction without upregulation in the Bip levels. However, astrocytes showed the nuclear activation of CHOP along with Bip upregulation, it was suggested that upregulation of proteins like Bip and protein-disulfide isomerase can protect the cells from apoptosis or delay apoptosis and different cell types responds to ER stress in different ways^68^. HERPUD1, an ER resident protein and a component of ERAD pathway has been shown to be upregulated in enterovirus 71 infections. HERPUD1 upregulation is associated with limiting virus replication by generating interferon mediated immune response^69^. It is unclear whether HERPUD1 upregulation is prevented during ZIKV infection in trophoblasts to ensure a productive infection by avoiding any innate immune response. However, further studies are required to confirm role of HERPUD1 in innate immune response evasion by flavivirus infection.

JNK is a master regulator of various physiological processes and are activated in response to stress and can modulate cell survival and cell death pathways^70^. Our data show that inhibition of JNK using small molecule led to the discovery that ZIKV-induced trophoblast apoptosis is a JNK-dependent process. JNK activation was also recently reported in mouse brain with ZIKV infection and in dengue virus infected human macrophages^71,72^. In our study, we found that JNK activation is critical in activating caspases to cause programmed cell death in ZIKV-infected placental trophoblasts. JNK activation is also well-established mediator that drives the cells undergoing ER stress into apoptotic pathway. Activation of JNK is known to enhance the translocation of Bax and Bak to the mitochondria, leading to Bax–Bak oligomerization and mitochondrial pore formation, which can result in activation of intrinsic pathway of apoptosis^73,74^.

ZIKV-induced trophoblast apoptosis was also protective with different inhibitors of ER stress pathway in a cell specific manner. Inhibition of IRE1α protects against apoptosis in third trimester derived placental trophoblasts. Selective inhibition of eIF2a dephosphorylation using salubrinal protects ZIKV infection-induced first trimester-derived trophoblast apoptosis. However, further studies are required to elucidate the protective role of ER stress inhibitors in suitable animal models of infection at various gestational ages. Expression profile of ZIKV viral entry cofactors varies in the placenta at different stages of gestation, for example, TIM1 is highly expressed in both JEG-3 and HTR-8/SVneo cells but Axl is not expressed in JEG-3 cells^16^. Viral entry is a crucial step for replication and there might be a time lag in the replication cycle based on the presence or absence of different viral entry cofactor expression, and this could in turn alter the survival dynamics of cells. Even though HTR-8/SVneo cells are derived from extravillous portion of the first trimester trophoblasts, recent studies show that they contain a population of trophoblasts as well as mesenchymal cells^75^, and further HTR-8/SVneo cells undergo epithelial mesenchymal transition which is a characteristic feature in placental development throughout the gestation period^76^. Further we also show reduction in *XBP1* mRNA splicing and viral load with JNK inhibition in ZIKV infected cells linking JNK activation and ER stress pathways. Treatment of salubrinal to ZIKV infected cells caused moderate decrease in *XBP1* mRNA splicing and viral load, respectively. Interestingly, inhibition of pan-caspase activity caused extensive *XBP1* mRNA splicing in the infected cells and increased viral load as the viral E proteins are trapped inside the cells and are unable to be released due to inhibition of apoptosis as previous reported^77^. A study found that inhibition of autophagy can limit ZIKV infection, this could be correlated to the fact that JNK also has a regulatory control on autophagy pathway and inhibition of JNK is known to inhibit autophagy^78–80^.

In conclusion, our data suggest that ZIKV infection induces ER stress and placental trophoblast apoptosis. Mechanistically, we have identified that JNK is a critical mediator of ZIKV-induced trophoblast apoptosis. Further studies are required to identify the molecular mechanism of JNK activation and other interacting partners with ZIKV infection and viral replication. Trophoblasts are the major cell type that interacts with maternal decidua and maternal blood circulation. Protective strategies against ZIKV infection in trophoblast can be crucial in preventing the transmission of ZIKV from the mother to the fetus.

## Materials and methods

### Cells

HTR-8/SVneo (HTR-8), normal human immortalized first-trimester placental trophoblast cells, and choriocarcinoma-derived third-trimester placental trophoblast cell lines (JEG-3 and JAR) were used. JAR and HTR-8 cells were cultured in DMEM containing 10% fetal bovine serum, 0.01% plasmocin. JEG-3 cells were cultured in MEM containing 10% fetal bovine serum, and plasmocin (0.01%). All the cells used the present study were obtained from ATCC and periodically tested for mycoplasma

### Antibodies

Primary antibodies against ZIKV E protein (#.GTX133314) from Gene Tex, Inc. CA, eIF2α (# 5324), p-eIF2α (# 3388), p-p38 (# 9211),p38 (# 9212), p-JNK(# 9251), JNK (# 9252), IRE1α(# 3294), CHOP (# 2895), Bip (# 3183), HDAC1(# 34589), HERPUD1(# 26730), GADD45 (# 46325), and BCl2 (# 15071) were purchased from Cell Signaling Technologies, MA. Phospho-IRE1α antibody (# ab48187) was obtained from abcam and Actin antibody (# A-5441) was from MilliporeSigma, MA. Rabbit HRP-conjugated secondary antibody (#111-035-144) and mouse HRP-conjugated secondary antibodies (# 715-035-150) were obtained from Jackson ImmunoResearch, PA. Alexa flour 488 conjugated anti-rabbit antibody (# A11008) and Alexa flour 594 conjugated anti-mouse antibody(# A-11032) were obtained from Invitrogen, CA.

### Viral strains and trophoblast infection

MR766 strain (originally isolated from sentinel monkey in Uganda), recombinant MR strain (r-MRV) of Zika virus is generated as described^21^, and PRVABC-59 strain (Asian lineage derived American strain from 2015 outbreak in Puerto Rico) were used in the present study. Trophoblasts were infected with 0.1−1 multiplicity of infection (MOI) in the infection media MEM (Gibco) containing 2% fetal bovine serum, streptomycin, penicillin, 7.5% sodium bicarbonate, 20 mM HEPES, 1 mM sodium pyruvate, and 1× nonessential amino acids and 0.01% plasmocin for 1–2 h. After virus adsorption, the media was replaced with DMEM or MEM containing 10% fetal bovine serum and 1% BSA.

### Characterization of apoptosis

Biochemical and structural markers of apoptosis like caspase 3/7 activity and percent apoptotic nuclear morphological changes, respectively. Caspase 3/7 (effector caspase) activity was measured by enzymatic fluorophore release (Apo-One) according to the manufacturer’s instructions (Promega, Madison, WI #G7791), and reported as fold change compared to vehicle treatment or mock infection, with experiments performed in quadruplicate. Cells were seeded in 24-well plates, infected with ZIKV for 48–72 h and percent apoptosis was quantified by characteristic nuclear morphology as described^22^, and visualized by treatment with the fluorescent DNA-binding dye, DAPI (4′,6-diamidine-2-phenylindole dihydrochloride) as described^22^. Briefly, cells were stained with 5 μg/ml of fluorescent DNA-binding dye, 4′,6-diamidine-2-phenylindole dihydrochloride (DAPI) for 20 min at 37 °C. Apoptotic nuclei (condensed, fragmented) were counted and presented as a percent of total nuclei. At least 100 cells were counted per well and experiments were performed in triplicate.

### Quantitative real time polymerase chain reaction

Cells were infected with ZIKV and the total RNA was isolated using TriZOL reagent (Thermo Scientific). Around 1–5 µg RNA from each sample was reverse transcribed to cDNA with random hexamers, RNAse OUT and Superscript II RNAse H (Invitrogen). CHOP mRNA and 18S rRNA were quantified using primers listed in Table 1 as described^23^.

**Table 1:** Primer details.

Primer	Forward primer	Reverse primer	Product length
XBP1	5′AAACAGAGTAGCAGC TCAGACTGC 3′	5′TCCTTCTGGGTAGAC CTCTGGGAG 3′	Unspliced forms’ ∼474 bp cleaved by the restriction endonuclease (Pst1) two products are around 296 bp and 183 bp; spliced forms lack restriction enzyme site ∼448 bp
GAPDH	5′AATCCCATCACCATC TTCCA 3′	5′TTCACACCCATGACG AAC AT 3′	∼194 bp
18srRNA	5′CGTTCTTAGTTGGTG GAGCG 3′	5′CGCTGAGCCAGT CAG TGTAG 3′	∼121 bp

### XBP1 splicing assay

Approximately 5 µg total RNA diluted 1:3 (JEG-3 and JAR cells) or 1:10 (HTR-8) was used for cDNA synthesis. Then it was subjected to PCR to amplify *XBP1* gene using the primer set (each 20 µM) as described^24^. Around 8 µl of the obtained PCR product was digested with 20 U of PstI (New England Bio labs) in 1 µl NEB buffer containing 100 mM NaCl, 50 mM Tris-HCl, 10 mM MgCl_2_, and 100 µg/ml BSA and incubation was done at 37 °C for 2 h. The restriction enzyme digested PCR product was electrophoresed in 2% agarose gel stained with ethidium bromide. The unspliced forms are around 474 bp and the restriction endonuclease digested products were 296 and 183 bp. The spliced forms lack restriction enzyme site so the bands are visualized around 448 bp. GAPDH was used as control and was amplified by the primers listed in Table 1.

### Western blot analysis

Cell lysates were prepared by adding 100 µl of lysis buffer containing 50 mM Tris pH 7.4, 150 mM NaCl, 1 mM EDTA, 1 mM DTT, 1 mM Na_3_Vo_4_, 1 mM PMSF, 100 mM NaF, and 1% Triton x-100. Cells were scraped, incubated for 40 min in ice and cell supernatant was prepared after 10,000 × *g* for 10 min of centrifugation. Protein estimation was done using modified Lowry method using Pierce 660 nm protein assay reagent (ThermoFisher Scientific). Around 30 µg protein wasseparated in a 10% polyacrylamide gel or 4–20% gradient gel and then transferred into nitrocellulose membrane. The membrane was blocked with either 5% skim milk powder in TBST or with 5% BSA in TBST. Primary antibody was used in 1:1000 dilution in 5% skim milk powder in TBST or with 5% BSA in TBST. Secondary antibody was used in 1:500 dilution. Washes of 10 min for three times were given after both primary and secondary antibody incubation. The blots were developed using Clarity Western ECL substrate or Clarity Max ECL substrate (Bio-Rad).

### Isolation of nuclear proteins

Cells were scraped in buffer containing 10 mM HEPES, 10 mM KCl, 0.1 mM EDTA, 0.1 mM DTT, 0.5% nonidet-P40 substitute (Sigma), and protease inhibitor and cell lysate were incubated for 10 min in ice. Cell lysates were centrifuged at 15,000×*g* for 3 min. The supernatant has cytoplasmic proteins and to the settled precipitated nuclear contents 50-100 µl of buffer B containing 20 mM HEPES, 0.4 M NaCl, 1 mM EDTA, 0.05 mM DTT, and 10% glycerol and proteinase inhibitor was added and incubated in ice with intermittent vortexing for 40 min and then centrifuged at 15,000×*g* for 5 min to collect the nuclear protein lysate in the supernatant.

### Immunofluorescence analysis

Placental trophoblast was infected with ZIKV, 0.1 MOI for 48–72 h. Infected cell media was aspirated and fixed with methanol and acetone (ratio of 1:1) and washed with PBS. Blocking was done using solution containing 1% BSA and 22.52 mg/ml glycine in PBST. Zika virus envelope protein (E protein) rabbit polyclonal was used at a dilution of 1:500 or CHOP mouse monoclonal antibody at a dilution of 1:400 was used and incubated overnight at 4 °C. After primary antibody incubation, cells were washed thrice with PBS with an interval of 5 mins each. Alexa flour 488/594 conjugated secondary antibody was added at a dilution of 1:1000 and kept in a shaker at room temperature for 2 h. After incubation, the cells were washed thrice in PBS, counter stained with DAPI (0.3 µM) for 10 min at 37 °C and washed with PBS. Images were taken using EVOS FL microscope (Thermo Fisher) or Nikon A1R-Ti2 confocal system.

### Statistical analysis

Analysis of variance (ANOVA) with post-hoc bonferroni corrections were employed for comparisons of multiple groups and Student’s *t*-test were performed for comparisons between two groups. *P* value < 0.05 was considered statistically significant.

## Supplementary information

Supplementary file

Fig S1

Fig S2
