# Supplementary material for: Zika virus infection induces endoplasmic reticulum stress and apoptosis in placental trophoblasts
Source: Cell Death Discov. 2021 Jan 26;7:24. doi: 10.1038/s41420-020-00379-8 (PMC7838309; doi:10.1038/s41420-020-00379-8)
Supplement: Supplementary file 1 — Supplementary file [file 41420_2020_379_MOESM1_ESM.docx]

**Zika virus infection induces endoplasmic reticulum stress and apoptosis in placental trophoblasts**

Philma Glora Muthuraj^1,2^, Prakash K. Sahoo^1,2^, Madison Kraus^1,2^, Taylor Bruett^1,2^, Arun S. Annamalai^2,3^, Aryamav Pattnaik^2,3^, Asit K. Pattnaik^2,3^, Siddappa N. Byrareddy^2,4,5^, Sathish Kumar Natarajan^1,2,6^*

^1^Department of Nutrition and Health Sciences, ^2^Nebraska Center for Virology, ^3^School of Veterinary Medicine and Biomedical Sciences, University of Nebraska-Lincoln; ^4^Department of Pharmacology and Experimental Therapeutics, ^5^Department of Genetics, Cell Biology and Anatomy, ^6^Department of Biochemistry and Molecular Biology, ^6^Child Health Research Institute, University of Nebraska Medical Center, Omaha, Nebraska, USA.

*Address for Correspondence: Sathish Kumar Natarajan, PhD

Assistant Professor

Department of Nutrition and Health Sciences

University of Nebraska-Lincoln

229 Filley Hall

Lincoln, NE 68583-0806

Tel: 402-472-7227; Fax: 402-472-1587

E-mail: [snatarajan2@unl.edu](mailto:snatarajan2@unl.edu)

ORCID: orcid.org/0000-0001-7491-8592

**Running title: ZIKV induces ER stress and apoptosis**

**Key words:** Placental barrier, congenital Zika syndrome, microcephaly, maternal-fetal transmission,

The project described was supported by Undergraduate Creative Activities & Research Experience (UCARE) to MK, and the University of Nebraska-Lincoln. The contents of this manuscript are solely the responsibility of the authors.

**Supplementary Fig.1. ZIKV infection results in downregulation of anti-apoptotic Bcl2.** Representative western blot showing anti-apoptotic Bcl_2_ is downregulated in JEG3 and HTR-8 cells, 24h and 16h post infection respectively.

**Supplementary Fig.2**. **CHOP expression in ZIKV infected placental trophoblasts**. CHOP mRNA expression is showing a trend towards increase around 24h post infection with 0.1 r-ZIKV MR strain in JEG-3 (A), JAR (B) and HTR-8 (C) compared to uninfected or mock-infected vehicle cells.
