# Supplementary figures and images for: Zika virus infection induces endoplasmic reticulum stress and apoptosis in placental trophoblasts
Source: Cell Death Discov. 2021 Jan 26;7:24. doi: 10.1038/s41420-020-00379-8 (PMC7838309; doi:10.1038/s41420-020-00379-8)

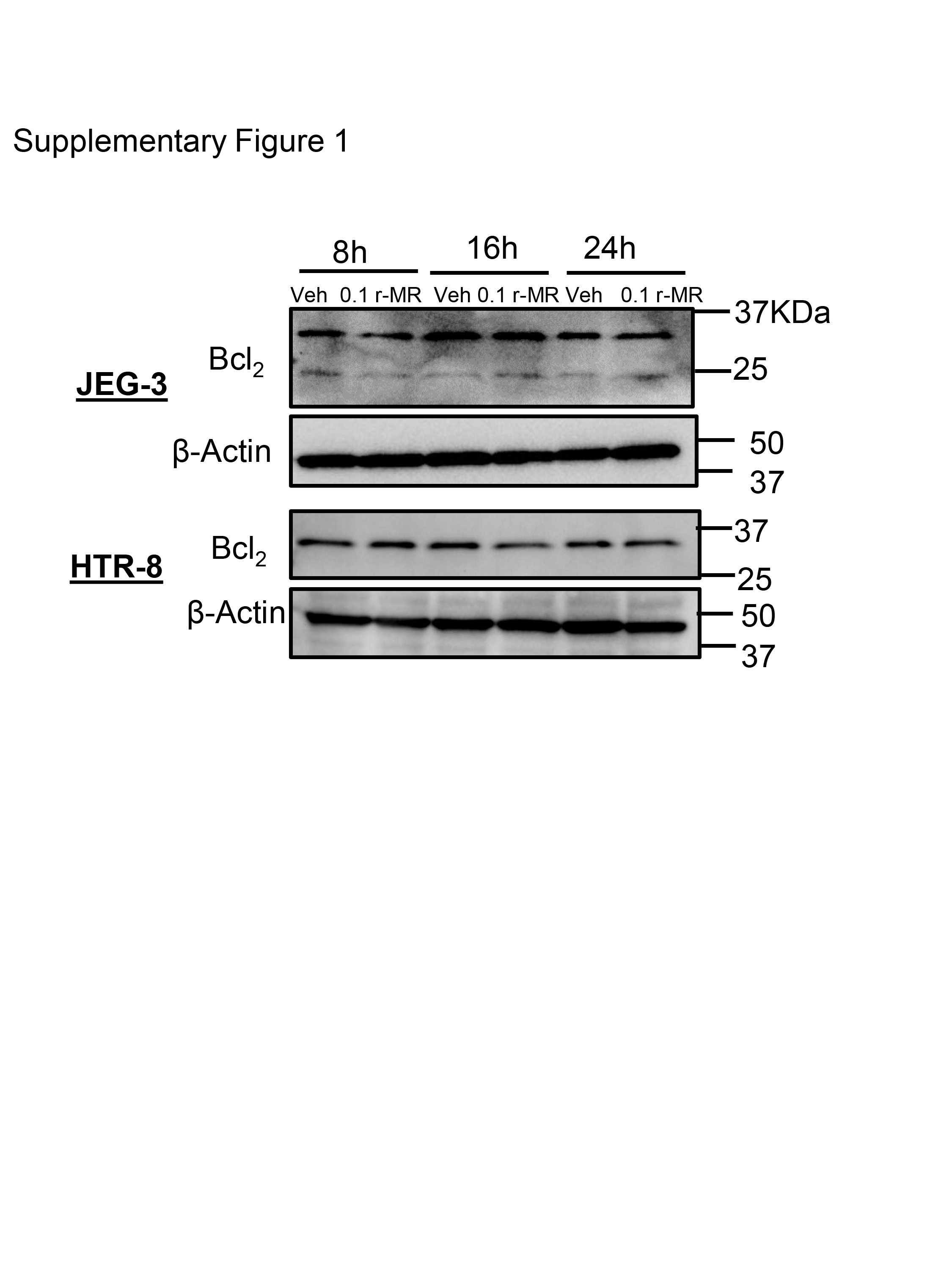

Supplement: Supplementary file 2 — Fig S1 [file 41420_2020_379_MOESM2_ESM.tif]

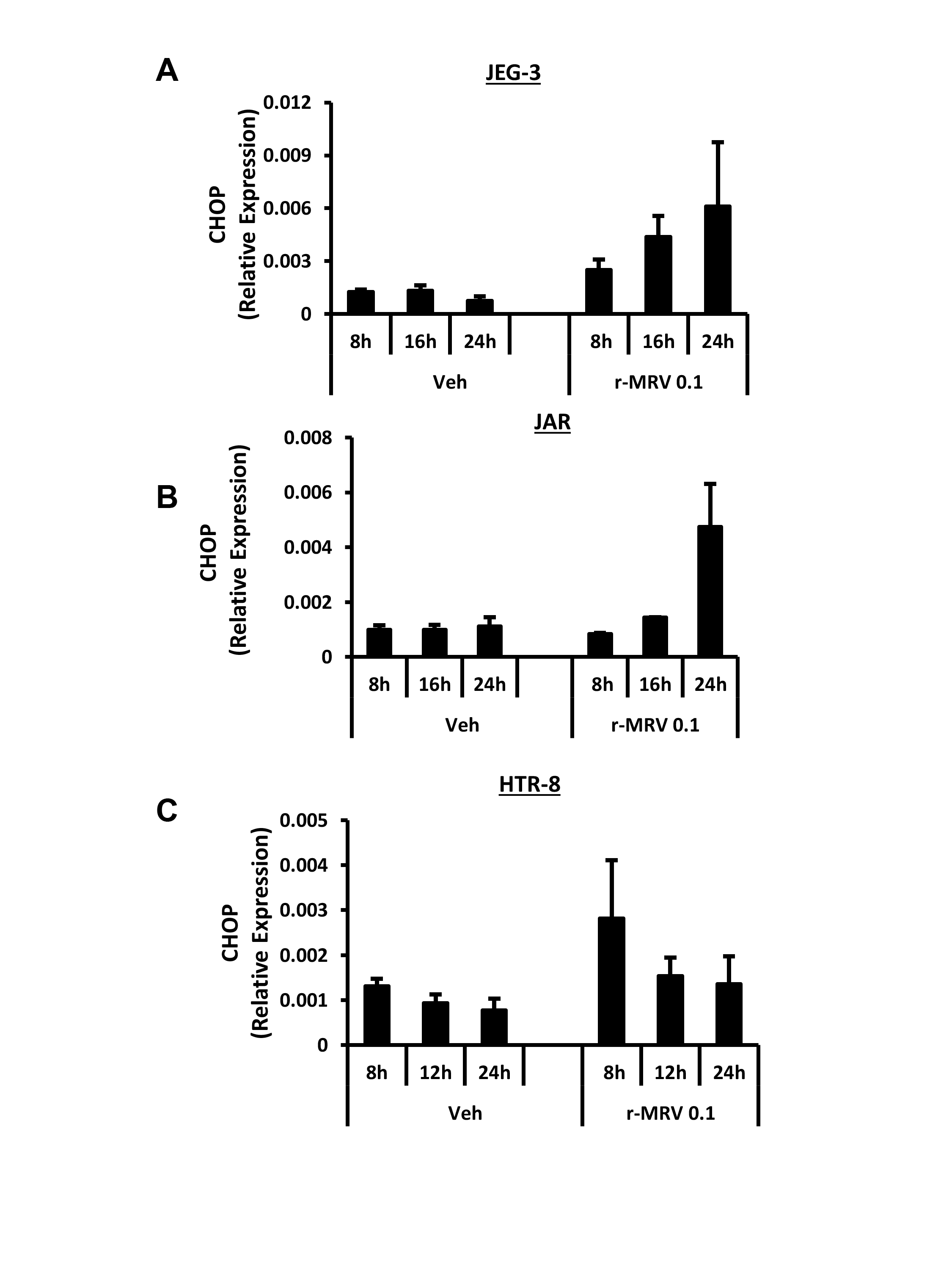

Supplement: Supplementary file 3 — Fig S2 [file 41420_2020_379_MOESM3_ESM.tif]
